# Live attenuated-nonpathogenic *Leishmania* and DNA structures as promising vaccine platforms against leishmaniasis: innovations can make waves

**DOI:** 10.3389/fmicb.2024.1326369

**Published:** 2024-04-03

**Authors:** Negar Seyed, Tahereh Taheri, Sima Rafati

**Affiliations:** Department of Immunotherapy and Leishmania Vaccine Research, Pasteur Institute of Iran, Tehran, Iran

**Keywords:** Leishmaniasis, vaccine, concomitant immunity, live attenuated vaccine, *Leishmania tarentolae*, antibiotic-free DNA vaccine

## Abstract

Leishmaniasis is a vector-borne disease caused by the protozoan parasite of *Leishmania* genus and is a complex disease affecting mostly tropical regions of the world. Unfortunately, despite the extensive effort made, there is no vaccine available for human use. Undoubtedly, a comprehensive understanding of the host-vector-parasite interaction is substantial for developing an effective prophylactic vaccine. Recently the role of sandfly saliva on disease progression has been uncovered which can make a substantial contribution in vaccine design. In this review we try to focus on the strategies that most probably meet the prerequisites of vaccine development (based on the current understandings) including live attenuated/non-pathogenic and subunit DNA vaccines. Innovative approaches such as reverse genetics, CRISP/R-Cas9 and antibiotic-free selection are now available to promisingly compensate for intrinsic drawbacks associated with these platforms. Our main goal is to call more attention toward the prerequisites of effective vaccine development while controlling the disease outspread is a substantial need.

## Graphical abstract

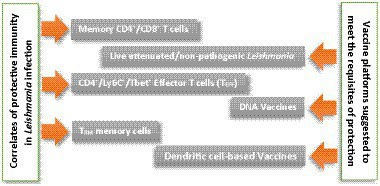

## Leishmaniasis: what we know

Neglected tropical diseases (NTDs) are diseases of poverty that impose a high socio-economic burden on more than 1 billion people worldwide, mainly in tropical and subtropical areas. Despite substantial progress in reducing the overall burden, the current road maps toward full eradication still need further improvement. Among the 20 different diseases in this category, leishmaniasis remains an unresolved threat to global health with an estimated annual rate of 700,000 to 1 million new cases leading to 20,000–30,000 deaths each year.^1^

Leishmaniasis is a vector-borne disease caused by the protozoan parasite of *Leishmania* genus and is a complex disease affecting mostly tropical regions of the world. The parasite with a dynamic genome has evolved to advantage a vector (sand fly) mediated transmission for successful disease establishment. Decades of labor-intensive hard work has partially deconvoluted the host-pathogen interaction which is partly controlled by different components deposited into the human skin through sand fly proboscis (Serafim et al., 2021). These include sand fly salivary proteins (Lestinova et al., 2017), parasite secretory gel (Sainz and de la Maza, 2014), exosomes carrying virulence factors (Dong et al., 2019, 2021), parasite associated double-stranded RNA viruses including *Leishmania* RNA Viruses (LRVs) (Rossi and Fasel, 2018) or non-LRV (Grybchuk et al., 2020) viruses inoculated by parasite or parasite exosomes, and even the gut microbiota of sand fly (Omondi and Demir, 2021). Sand fly saliva, is composed of different immunomodulatory components among which chemotactic factors for neutrophils are recently characterized (Guimaraes-Costa et al., 2021), LRVs trigger TLR-3 for pro-inflammatory cytokine production (Hartley et al., 2012), gut microbiota induces IL-1b production via inflammasome activation (Dey et al., 2018) and parasite dependent factors induce Toll-like receptors (TLR) including TLR-2, TLR-4, TLR-7, and TLR-9 (Masoudzadeh et al., 2020a,b). The cumulative consequence of this inflammatory response is the massive neutrophilic recruitment to the bite site early after infection (Peters et al., 2008). While some parasites are killed by neutrophils like *Leishmania (L.) amazonensis*, others are able to survive by skipping the killing mechanisms like *L. major* (Passelli et al., 2021). Depending on the parasite species, host related immune response and genetic background, neutrophil recruitment to the bite site can then affect the outcome of the infection (Peters and Sacks, 2009). Although neutrophil function has shown protective in some experimental leishmaniasis (de Souza Carmo et al., 2010; Carlsen et al., 2015), in other experiments, massive neutrophil recruitment has correlated with more prominent lesions by providing a permissive environment for disease establishment (Ronet et al., 2019; Chaves et al., 2020).

## Vaccine development and current gaps in our knowledge

Unfortunately, despite the high burden of the disease around the world (more than 1 billion people live in areas endemic for leishmaniasis and are at risk of infection with an annual estimation of 30,000 new cases of VL and more than 1 million new cases of CL), there is no vaccine available for human use. All we know so far is that full protection against *Leishmania* parasite-induced infection generally requires CD4^+^-Th1 response. Therefore, extensive effort has been made to generate memory Th1 response advantaging so many different vaccine platforms with promising experimental results (Costa et al., 2011; Dinc, 2022).

It was not until recently when it was realized that vector derived compounds abrogate vaccine efficacy in sandfly-derived infection (Peters et al., 2009). This has been concluded from the comparisons made between leishmanization and different killed or multiprotein subunit formulations (already accepted as promising candidates) taking into account the sand fly versus needle challenge (Seyed et al., 2018). ALM-CpG (Autoclaved *Leishmania major* adjuvanted with CpG oligonucleotide) as killed vaccine (Peters et al., 2009) in addition to KSAC-GLA.SE [recombinant protein adjuvanted with stable emulsion (SE) formulation of glucopyranosyl lipid A (GLA)] and L110F-GLA.SE as poly-protein vaccines (Peters et al., 2012) have been compared in protection efficacy to leishmanization in natural sand fly challenge of C57BL/6 mice. Although all these formulations potentially protect against needle *Leishmania* challenge when it comes to parasite load, but none of them are able to emulate the protection conferred by leishmanization after sand fly challenge in C57BL/6 mice. Of note, the same KSAC-GLA-SE formulation examined in BALB/c mice conferred protection against *Leishmania* major transmitted by sand fly bites (regarding lesion size and parasite burden) due to a strong immune response to *Leishmania* antigens by memory T cells after sand fly transmission of the parasite (Gomes et al., 2012). The sustained and massive neutrophilic recruitment up to 28 days post sand fly infection in C57BL/6 mice was the major complicating factor compared to needle challenge which inversely dampens the vaccine’s protection potential. It was postulated that leishmanization potentially modulates these early inflammatory events post sand fly challenge by a rapid and robust IFN-γ mediated intervention. It was recently unraveled that this rapid and robust IFN-γ response cannot be provided by memory Th1 cells at the time of infection since they are activated and proliferate at later time points (Seyed and Rafati, 2021). Generally speaking, this means that for achieving a potential vaccine against leishmaniasis, we have to recognize the correlates of protective immunity besides Th1 memory T cells.

## “Ongoing chronic infection” is the key to vaccine development against vector-born leishmaniasis

Among the different platforms examined so far (Kaye et al., 2021; Volpedo et al., 2021), leishmanization remains the only vaccine formulation effective against *Leishmania* infection in endemic areas (although not approved for general use in humans) (Pacheco-Fernandez et al., 2021). The lessons learned from leishmanization has led to the “concomitant Immunity” concept which means “ongoing chronic infection” (Sacks, 2014). This primary subclinical chronic infection with life-long persistent parasites at low levels after healing, induces major subsets of effector and memory T cells which play a critical role early after sand fly bite at secondary infection site. The immune correlates associated with “concomitant Immunity” include skin-resident memory T cells (T_RM_) and effector Ly6C^+^ T cells (T_EFF_) besides Th1 central (T_CM_) and effector memory (T_EM_) cells (Hohman and Peters, 2019; Seyed and Rafati, 2021). Ly6C^+^ T_EFF_ cells (Peters et al., 2014), and T_RM_ cells (Glennie and Scott, 2016; Scott, 2020) play an indispensable role in protection against sand fly deposition of the parasite. They promote a non-permissive environment for parasite growth in inflammatory monocytes recruited to the bite site by providing early (within hours) and robust local IFN-γ. This means that timing is everything. Hohman et al. clearly demonstrated that permissive phagocytic host cells’ activation by pre-activated Ly6C^+^ T_EFF_ cells is the pre-requisite for Th1 induced protection (Hohman et al., 2021). Otherwise, delayed Th1 central memory cell activation does not confer full protection after the permissive environment is established by skin-deposited immunomodulatory sand fly components (Hohman et al., 2021). These cells are well characterized in mouse models and we still need to identify the human counterparts. Mouse T_EFF_ cells which rapidly accumulate at the bite site, are ready to take action without proliferation and produce large amounts of IFN-γ to provide a timely and robust response. These cells are very much antigen dependent and disappear in the absence of antigen. T_RM_ cells instead are non-circulating tissue resident memory cells which mediate CCL2 dependent recruitment of inflammatory monocytes highly expressing MHCII molecules and producing active anti-leishmanial metabolites mainly nitric oxide and reactive oxygen derivatives.

## Not all vaccine platforms can provide concomitant immunity

As described, the prominent characteristic of leishmanization-induced protection is the persistence of parasite after cure (Belkaid et al., 2001). In other words, long-lasting antigen presentation remains the key challenging point for the alternative platforms other than leishmanization and mainly the subunit vaccines. In the absence of distinguished T_RM_ and Ly6C^+^ T_EFF_ cells, permissive phagocytic cells lead to parasite propagation and disease establishment early after sand fly bite. Therefore, for a vaccination strategy to succeed, pre-activated Ly6C^+^ T_EFF_ (Hohman and Peters, 2019) and T_RM_ (Glennie et al., 2015) cells are necessary without which the formulation will most likely fail in field trials even in the presence of memory T cells. Although it still remains elusive how to best generate T_RM_ and Ly6C^+^ T_EFF_ cells by vaccination, live attenuated parasites and subunit multivalent DNA vaccines are here suggested as alternatives of leishmanization for long term antigen presentation. We have discussed the advantages and have suggested the dendritic-cell based vaccines in another paper (Seyed et al., 2018). Here, we summarize the evidence why live attenuated parasites and subunit multivalent DNA vaccine strategies can be attractive for more intensive investigation. Several innovative approaches including reverse genetics, CRISPR/Cas9 (clustered regularly interspaced palindromic repeats) technology and antibiotic free selection are now available to overcome the intrinsic drawbacks of these platforms and make waves in the future of vaccination against leishmaniasis. The rest of the vaccination platforms, although examined in experimental models or even in clinical trials, remain to address the question whether they can provide a long-lasting antigen presentation or not.

## Live attenuated parasite vaccines most likely can replace leishmanization

Live attenuated vaccines of different pathogens have successfully eradicated many intracellular pathogens such as poliovirus and smallpox (variola virus) and have controlled many others such as measles, mumps and rubella viruses (Plotkin, 2014). Mimicking the normal course of the infection with natural molecular patterns to induce innate immunity, complete set of antigens delivered and above all, subclinical infection which lasts longer than any protein subunit formulation, are among the major factors which prioritize the live vaccines (Silvestre et al., 2008). These traits are in-line with the *Leishmania* vaccination goal and has led to the intensive effort for attenuating different parasite species. So far various genes have been explored as targets of attenuation both in cutaneous (Zabala-Penafiel et al., 2020) and visceral (Pandey et al., 2020) leishmaniasis with the aim of generating long-lived non-virulent parasites unable to revert back to the wild type parental parasite. Among others, *lpg*2^−/−^ *L. major* has shown a long persistence up to 2 years in BALB/c mice without pathology (Spath et al., 2003). This mutant strain is cleared easily within macrophages and is assumed to persist in cells other than macrophages *in vivo*. This model increased hopes for a protective vaccine with encouraging results in mouse models (Uzonna et al., 2004) but the dreams were ruined when the compensatory mutants (*lpg*2^−/−^ REV) were detected (Spath et al., 2004). This means that a major concern for a parasite with a dynamic genome is the ability to compensate for the mutant gene and revert back into the pathogenic parasite which is the top concern of regulatory authorities regarding live attenuated pathogens. Thus, in addition to the persistence without pathology, choosing for virulence genes which cannot be compensated by other genes is of paramount importance in generating live attenuated *Leishmania* strains.

Recently, genetically modified live attenuated Centrin^−/−^ (Cen^−/−^) *L. donovani* (Bhattacharya et al., 2016), *L. major* (Zhang et al., 2020) and *L. mexicana* (Karmakar et al., 2022) mutants have generated promising results as vaccine candidates (Volpedo et al., 2022). Centrin, a calcium-binding cytoskeletal protein, is concerned with the basal body duplication and segregation in lower eukaryotes (Salisbury, 2004). The lack of basal body formation and cytokinesis leads to apoptosis and G2/M phase cell arrest. Therefore, Centrin mutation inhibits amastigote proliferation in macrophages leading to immunological clearance as suggested (Selvapandiyan et al., 2001, 2004). LmCen^−/−^ parasites represent an equally effective but safer alternative to leishmanization for the protection induced against *L. major* transmitted by sand fly bite (Silvestre et al., 2008) and also cross protection against more virulent *L. donovani* (Karmakar et al., 2022). In particular, mice immunized with LmCen^−/−^ parasites compared to mice healed from *L. major* leishmanization, have generated a significantly higher pro-inflammatory immune response, characterized by CD4^+^CD44^high^Ly6C^+^T-bet^+^ IFN-γ^+^ effector T cells (Zhang et al., 2020) and tissue resident memory (T_RM_) T cell responses (Ismail et al., 2022). This is the first report of a live attenuated formulation that enables Ly6C^+^ T_EFF_ and T_RM_ induction without a compensatory reversion. Owing to the fast-growing CRISPR/Cas9 gene editing technology, LmCen^−/−^ mutant is now at the front line to initiate human clinical trials since the parasite lacks antibiotic resistance genes which is another top concern of regulatory authorities about genetically modified pathogens (Zhang et al., 2020). CRISPR/Cas9-mediated gene targeting and editing facilitates deletion of essential genes (either single or multigene families) to observe the functional and phenotypic effects on living cells in a time-dependent manner (Zhang and Matlashewski, 2015; Adaui et al., 2020). The fast development of this technology will certainly accelerate the production of knockout mutants in *Leishmania* instead of homologous recombination-based gene replacement making more live attenuated parasites available in near future (Singh et al., 2022; Moreira et al., 2023).

With the advent of reverse instead of forward genetics and with complete parasite genomes now available due to new technologies for whole genome sequencing, new horizons have appeared. Non-pathogenic lizard-isolated *Leishmania (L.) tarentolae* is a precious tool with the whole genome sequence now available. In a study by Azizi et al. searching for virulence factors in *L. tarentolae*, it was realized that the lack of pathogenicity is not LPG3, CPB, GP63 and Amastin dependent (Azizi et al., 2009) which reflected the importance of other genes either unique to *L. tarentolae* or missing from this species for the nonpathogenic potential of this lizard parasite. Later, the whole genome of this non-pathogenic parasite was sequenced and compared to *L. major, L. infantum* and *L. braziliensis* by Reymond *et al* (Raymond et al., 2012). This “subtractive genomics” study identified 95 predicted coding sequences unique to *L. tarentolae* and 250 genes present in the pathogenic species but absent in *L. tarentolae* which are mainly associated with the amastigote stage. This explained in part why *L. tarentolae* proliferates less well in human macrophages and why these parasites are mostly reported as free organisms in the lizards. Fortunately, reverse genetics tools can unveil new virulence factors out of these unique or missing genes associated with non-pathogenic *L. tarentolae*. Duncan et al. have described these reverse genetics tools in different categories (Duncan et al., 2017) including tools for assessing if *Leishmania* genes are essential for cellular proliferation like plasmid shuffle (McCall et al., 2015), DiCre (dimerizable Cre) recombinase (Madeira da Silva and Beverley, 2010; Schindler et al., 2015) and CRISPR/Cas9 (Zhang et al., 2017) or tools for regulating gene expression such as RNAi (de Paiva et al., 2015), Tetracycline inducible gene expression (Ishemgulova et al., 2016) or “DiCre recombinase inducible gene expression” (Santos et al., 2017). Tools for endogenous tagging and regulating protein levels are also introduced such as “endogenous tagging” and “conditional protein destabilization” (Damerow et al., 2015; Dean et al., 2015). Fortunately, all of these new technologies are applicable for manipulating pathogenic *Leishmania* genomes and/or proteomes with the hope of finding novel virulence factors in comparison to non-pathogenic strains such as *L. tarentolae.*

## Non-pathogenic *Leishmania tarentolae* parasite can take over as vaccine

*Leishmania tarentolae* has also been investigated as vaccines against human pathogenic *Leishmania* species (Bandi et al., 2023). After Breton et al. showed that intra-peritoneal administration of live *L. tarentolae* in BALB/c mice induces a Th1 pathway with significant protection against *L. donovani* infectious challenge (Breton et al., 2005), non-engineered live *L. tarentolae* promastigotes were assayed as candidate vaccines adjuvanted with CpG oligonucleotides (Keshavarzian et al., 2020), or chitin microparticles against *L. major* (Haghdoust et al., 2022; Noroozbeygi et al., 2023). Others employed genetically modified strains of *L. tarentolae*, engineered for overexpression of antigens from human pathogenic *Leishmanias* (Saljoughian et al., 2013; Topuz Ata et al., 2023) or antigens from the vector saliva (Katebi et al., 2015; Lajevardi et al., 2022). These studies generally showed that *L. tarentolae* induces protection in animal models against pathogenic species, including *L. infantum* and *L. major* (Mendoza-Roldan et al., 2022). We still need to further address the induction of Ly6C^+^ T_EFF_ and/or T_RM_ cells by this platform. *L. tarentolae* is able to enter human phagocytic cells and differentiate into amastigote like forms. However, there is no clear evidence for their efficient replication within macrophages (Raymond et al., 2012). Therefore, the top priority is to determine whether the parasite persists *in vivo* and if yes for how long and in which cells. A recently published paper has detected *L. tarentolae* in blood samples from dogs and lizards in an endemic area of southern Italy and also in sand flies. Amazingly, at the cytology of lizard blood, *Leishmania* spp. amastigote-like forms were detected in erythrocytes (Mendoza-Roldan et al., 2021).

## DNA vaccines are among the subunit strategies that most likely provide durable immunity

With the advent of molecular genetics and recombinant technologies, DNA-based vaccines (also called genetic vaccines) which advantage the bacterial plasmid constructs, moved to the frontline to pioneer for vaccine design against infectious diseases (Gary and Weiner, 2020). They are more stable in biological systems and induce strong and long-lasting antigen-specific cell-mediated immune responses due to the sustained *in vivo* expression of antigen, efficient antigen presentation and the presence of in-built innate immunity-stimulatory CpG motifs as adjuvant (McCluskie et al., 2000; Lee et al., 2018). Plasmids are small circular structures engineered to accommodate genes from different organisms for *in vivo* production. The plasmid backbone is composed of eukaryotic sequences for protein production in eukaryotes and also prokaryotic sequences for replication and selection in laboratory production steps.

Remarkable advantages of DNA vaccines include induction of a prolonged cellular and humoral immunity, ease of large-scale production, adaptability to encode several antigens and above all self-adjuvanicity. DNA vaccines are currently licensed for several animal diseases (Kutzler and Weiner, 2008). In many cases of cancer, they are also used as therapeutic vaccines and they have even reached the second and third phases of human preventive vaccine in clinical trials (Lopes et al., 2019). However, the potential of DNA vaccines has not been realized due to the poor cellular uptake of DNA *in vivo*, resulting in poor immunogenicity. Innovative delivery systems are now available to improve cellular uptake and consequently the immunogenicity (Lim et al., 2020; Ho et al., 2021).

In *Leishmania* vaccine research, DNA vaccines have made an important contribution because of long-lasting Th1 immunity conferred against the parasite. One of the major factors in sustained Th1 response differentiation is IL-12 cytokine produced by dendritic cells which is well provided by DNA vaccination instead of protein plus IL-12 (Gurunathan et al., 1997). In 2001, Mendez et al. compared the DNA vaccination versus protein/recombinant IL-12 conferred protection in low dose infectious challenge with *L. major* (intradermal inoculation of 100–1,000 metacyclic promastigotes). Three months post-immunization a potential sustained protective immunity due to DNA vaccination versus a partial protection due to protein/IL-12 vaccination was observed (Mendez et al., 2001). The durable protection in mice vaccinated with DNA was associated with the recruitment of both CD8^+^ and CD4^+^ T cells to the site of intradermal challenge and with IFN-γ production by CD8^+^ T cells in challenge draining lymph nodes. This is attributable to the sustained IL-12 production besides low levels of persistent antigen produced by DNA structures (Gurunathan et al., 2000). Although these results might well correspond with the long-lived memory T cells which potentially protect against sand fly components-free challenge, but are invaluable respecting the more sustained antigen presentation by DNA structures compared to naked-recombinant protein formulations.

After introducing the role of sand fly proteins in protection against sand fly transmitted infection (Kamhawi, 2000; Kamhavi et al., 2018), other groups evaluated the durability of protection conferred against more natural infection. Salivary Gland Homogenates (SGH) of the vector was co-injected with intradermal low dose parasite after DNA vaccine immunization encoding *Phlebotomus (Ph.) papatasi* Sp15 protein (a 15 kilodalton protein). Three months post booster immunization, the protection was quite comparable to that achieved 2 weeks post vaccination with a significant reduction in both lesion size and parasite number (Valenzuela et al., 2001). Although this group never evaluated the biomarkers of long-lasting protection (Ly6C^+^ T cells and T_RM_ cells) required for protection against SGH-induced inflammatory response, but the results raised the hope.

Recently Davarpanah et al. advantaged the *Lactococcus lactis* based-DNA transfer technology to evaluate the protection against *Ph. papatasi* SGH co-injected with *L. major* parasite. Six months following immunization with *Ph. papatasi* Sp15 expressing *L. lactis*, the lesion size and parasite number was significantly lower in Sp15 vaccinated animals as compared to control groups due to a Th1 polarized immune response (Davarpanah et al., 2020). Again, the SGH-required protection biomarkers were not evaluated, however a significant protection almost comparable to the early detected protection (within 2 weeks post challenge) could be a good sign.

Luise et al. recently conducted an experiment to evaluate the claim that intradermal (i.d.) immunization or scarification effectively generates T_RM_ cells in the skin (Sangare et al., 2009). To address this issue, they have compared the generation of skin-resident T cells and protection against *L. major* induced cutaneous leishmaniasis following intra-muscular and intra-dermal DNA immunizations routes. A plasmid DNA encoding *Leishmania* phosphoenolpyruvate carboxykinase was injected intradermaly by electroporation and also intramuscularly. As indicated, intradermal immunization better protected against *L. major* challenge. Of note, the protection level was comparable to the leishmanized-immune mice previously infected with parasites both in lesion size and parasite burden. They concluded that the intradermal route may be most efficient at generating T_RM_ cells and protection against *Leishmania* parasites (Louis et al., 2019). This robust document indicating the T_RM_ generation following DNA immunization by intradermal route, further pushes the DNA vaccine strategy to the top of the list of candidate vaccine formulations against leishmaniasis.

## Antibiotic-free plasmids might bring the DNA vaccines back into focus

Despite the fact that specific complications such as DNA insertion into the chromosome and autoimmunity or tolerance have not been reported (Liu, 2011), unfortunately no DNA-based vaccine is approved for human infections including Leishmaniasis. Although the immunogenicity of DNA vaccines as a major concern is under intensive investigation (extensively reviewed elsewhere) and major improvements have been achieved (Li and Petrovsky, 2016; Suschak et al., 2017), but this is not the real reason. In fact, the most important reason is the prokaryotic backbone of the plasmid which are advantaged in plasmid production steps only. Following DNA entry into the eukaryotic host cell, only the eukaryotic regions including promoter, enhancer sequences, and polyadenylation sequences are used, and the prokaryotic region has no use at all. Instead, these prokaryotic sequences can cause serious problems after transfection which are discussed as follows (Li and Petrovsky, 2016; Eusebio et al., 2021):

First, the presence of an antibiotic resistance gene can spread antibiotic resistance to other bacteria in the vaccinated person through horizontal transfer. Many plasmid structures use aminoglycoside resistance genes such as kanamycin and neomycin which have extensive clinical uses. Therefore, there is a risk of developing resistance to these antibiotics. Some other structures use beta-lactam resistance genes, such as ampicillin. Antibiotics remnants after the *in vitro* purification process, can lead to a severe anaphylactic reaction in susceptible individuals (Williams, 2013). Second, antibiotic resistance in large-scale culture of parasites reduces the plasmid production yield resulting from metabolic pressure on bacterial cells, because the antibiotic in the culture medium induces constitutive expression of the resistance gene which slows down the growth rate of bacteria (Rozkov et al., 2004; Mairhofer et al., 2010). Third, the prokaryotic backbone can interfere with the expression of the gene while inside the eukaryotic cells. After entering the eukaryotic cell, the prokaryotic region becomes predominantly heterochromatin, which can spread to the target gene region. In addition, prokaryotic regions may have cryptic promoters that produce small RNA sequences in eukaryotic cells and block the expression of encoded gene’s mRNA (Lu et al., 2012; Gracey Maniar et al., 2013). Finally, first-generation plasmids used for vaccines or immunotherapy, such as pcDNA, are relatively large in size, and much of this large structure belongs to prokaryotic regions that interfere with the efficiency of transfection. Comparison has evidenced the relation between the smaller DNA structure and the higher transfection efficiency. Although methods such as electroporation, gene guns, or liposomes and lipid nanoparticles help increase transfer from the membrane into the cell, the large size of the plasmid slows down the transfer from the cytoplasm to the nucleus (Lukacs et al., 2000; Yin et al., 2005; Rosazza et al., 2011).

New generation of plasmids devoid of antibiotic resistance marker with shorter prokaryotic backbones are now available to overcome these drawbacks (Vandermeulen et al., 2011). They are either based on complete elimination of the prokaryotic sequences to generate small-sized minicircles (which are not further plasmids) or selection systems other than antibiotic resistance. The latter include complementation of auxotrophic strains by suppressive tRNAs (Marie et al., 2008, 2010; Bakker et al., 2019), toxin–antitoxin systems (Bukowski et al., 2011), operator–repressor titration (Cranenburgh et al., 2001; Mwau et al., 2004; Ramos et al., 2009), RNA markers including RNA out (Luke et al., 2009) and RNA I (Pfaffenzeller et al., 2006) systems, overexpression of a growth essential gene (Goh and Good, 2008) and enzyme-inhibitor ratios (Alcolea et al., 2019). These are altogether known as marker-free plasmids.

Minicircles were designed in 1997. Using homologous recombinant technology, the prokaryotic parts were removed from the parental plasmid after the completion of the *in vitro* amplification step (Darquet et al., 1997). The use of these structures with a prokaryotic region below 100 base pairs, manipulated either before inoculation or within the cells after transfection, has generated promising results in vaccine studies of cancer (Pang et al., 2017), Hepatitis B virus (Li et al., 2016), HIV (Wang et al., 2014), Newcastle Disease (Jiang et al., 2019) and *Listeria monocytogenes* (Dietz et al., 2013). In all cases, an elevated protection level following long-term expression of the antigen has been observed when compared to the first-generation plasmids (Munye et al., 2016). However, the purification of these small-sized constructs is much more labor intensive and complicated than the conventional plasmids. Although newer systems suggest the removal of the prokaryotic part *in vivo* (Jiang et al., 2019), this can leave a mixture of the parental plasmid and minicircles within the same cell.

Besides Minicircles, marker-free plasmids are also under investigation as vaccine or non-viral gene therapy tools. Among the many different antibiotic-free selection systems, RNA-based methods are currently more attractive. Recently, in a study published by Suschak, the effect of DNA vaccine against Ebola and against Venezuelan Equine Encephalitis Virus (VEEV) was compared using pWRG7077 plasmids and an RNA-OUT system along with immunostimulatory sequences (CpG and Immunostimulatory RNA) and it was determined that an RNA-OUT system as in the case of VEEV, the vaccine had a similar protective effect and in the case of the Ebola vaccine, it had a better effect than the pWRG7077 plasmid (Suschak et al., 2020).

Other selection systems are also under investigation with promising results. Alcolea et al. have examined the Th1 response induction levels against canine leishmaniasis using pPAL-LACK vector (an enzyme-inhibitor system). The protection was comparable to recombinant vaccinia virus in combination with standard mammalian expression plasmid vectors. The pPAL vector (3,899 bp in length) contains the cytomegalovirus enhancer and promoter for expression in mammalian cells and the *E. coli fabI* chromosomal gene as a selectable marker. The pPAL plasmid contains the essential elements for manipulation and expression of any cloned DNA sequence in prokaryotic and mammalian cells using an *E. coli* endogenous gene as a selectable marker, which also provides a long CpG island. This antibiotic resistance-free plasmid is a vaccine vector actively participating in protection against canine leishmaniasis, and may be potentially tested as a vaccine vector with other antigens against different pathogens (Alcolea et al., 2019).

## Concluding remarks

Leishmaniasis is a vector-borne parasitic infection with an unresolved complex host-pathogen interaction. That is why a protective vaccine is missing for human use despite labor intensive hard work to reach the point. However, vaccination is still a hope and not hype referring to the field observations like asymptomatic infection in endemic areas. Apparently, the more we understand host pathogen interaction, the closer we get to a protective vaccine.

Knowing that the efficacy of the Th1 memory protection is abrogated following sand fly transmission of parasite, the vaccine options are more restricted particularly in non-endemic areas. Live attenuated, live nonpathogenic and DNA vaccines seem the best choices as long as they persist in the body and continue producing the pertinent antigens. Therefore, we can think of these choices in endemic areas where the immunization occurs within a few months before sand fly biting season. For sure, intradermal immunization and intradermal low dose sand fly challenge are of paramount importance respecting the T_EFF_ and T_RM_ cells for effective vaccine design. Hopefully, novel innovative approaches such as CRISPR/Cas9 technology and antibiotic-free plasmids will improve intrinsic drawbacks associated with live attenuated parasites or DNA vaccines.

Alternatively, raising neutralizing antibodies against sand fly derived factors could be applicable both for endemic and non-endemic areas. Of note some sand fly derived proteins are identified to recruit neutrophils to the bite site. Therefore, neutralization of these proteins by vaccination and antibody generation could be a good choice to restrict sand fly saliva mediated neutrophil recruitment. Albeit, the pre-requisites for the long-lived plasma cell and memory B cell generation remain to be fully addressed and are still under intensive investigation.

## Author contributions

NS: Conceptualization, Supervision, Writing – original draft. TT: Writing – review & editing. SR: Writing – review & editing.

## Footnotes

^1^ https://iris.who.int/bitstream/handle/10665/363155/9789240052932-eng.pdf

## References

[ref1] Adaui V. Krober-Boncardo C. Brinker C. Zirpel H. Sellau J. Arevalo J.. (2020). Application of CRISPR/Cas9-based reverse genetics in *Leishmania braziliensis*: conserved roles for HSP100 and HSP23. Genes (Basel) 11:1159. doi: 10.3390/genes11101159, PMID: 33007987 PMC7601497

[ref2] Alcolea P. J. Alonso A. Larraga V. (2019). The antibiotic resistance-free mammalian expression plasmid vector pPAL for development of third generation vaccines. Plasmid 101, 35–42. doi: 10.1016/j.plasmid.2018.12.002, PMID: 30529129

[ref3] Azizi H. Hassani K. Taslimi Y. Najafabadi H. S. Papadopoulou B. Rafati S. (2009). Searching for virulence factors in the non-pathogenic parasite to humans Leishmania tarentolae. Parasitology 136, 723–735. doi: 10.1017/S0031182009005873, PMID: 19416551

[ref4] Bakker N. A. M. de Boer R. Marie C. Scherman D. Haanen J. Beijnen J. H.. (2019). Small-scale GMP production of plasmid DNA using a simplified and fully disposable production method. J. Biotechnol. 306:100007. doi: 10.1016/j.btecx.2019.100007 34112376

[ref5] Bandi C. Mendoza-Roldan J. A. Otranto D. Alvaro A. Louzada-Flores V. N. Pajoro M.. (2023). *Leishmania tarentolae*: a vaccine platform to target dendritic cells and a surrogate pathogen for next generation vaccine research in leishmaniases and viral infections. Parasit. Vectors 16:35. doi: 10.1186/s13071-023-05651-1, PMID: 36703216 PMC9879565

[ref6] Belkaid Y. Hoffmann K. F. Mendez S. Kamhawi S. Udey M. C. Wynn T. A.. (2001). The role of interleukin (IL)-10 in the persistence of Leishmania major in the skin after healing and the therapeutic potential of anti-IL-10 receptor antibody for sterile cure. J. Exp. Med. 194, 1497–1506. doi: 10.1084/jem.194.10.1497, PMID: 11714756 PMC2193677

[ref7] Bhattacharya P. Dey R. Dagur P. K. Joshi A. B. Ismail N. Gannavaram S.. (2016). Live attenuated *Leishmania donovani* Centrin Knock out parasites generate non-inferior protective immune response in aged mice against visceral Leishmaniasis. PLoS Negl. Trop. Dis. 10:e0004963. doi: 10.1371/journal.pntd.0004963, PMID: 27580076 PMC5007048

[ref8] Breton M. Tremblay M. J. Ouellette M. Papadopoulou B. (2005). Live nonpathogenic parasitic vector as a candidate vaccine against visceral leishmaniasis. Infect. Immun. 73, 6372–6382. doi: 10.1128/IAI.73.10.6372-6382.2005, PMID: 16177308 PMC1230936

[ref9] Bukowski M. Rojowska A. Wladyka B. (2011). Prokaryotic toxin-antitoxin systems--the role in bacterial physiology and application in molecular biology. Acta Biochim. Pol. 58, 1–9. doi: 10.18388/abp.2011_2278, PMID: 21394325

[ref10] Carlsen E. D. Jie Z. Liang Y. Henard C. A. Hay C. Sun J.. (2015). Interactions between neutrophils and *Leishmania braziliensis* amastigotes facilitate cell activation and parasite clearance. J. Innate Immun. 7, 354–363. doi: 10.1159/000373923, PMID: 25766649 PMC4485586

[ref11] Chaves M. M. Lee S. H. Kamenyeva O. Ghosh K. Peters N. C. Sacks D. (2020). The role of dermis resident macrophages and their interaction with neutrophils in the early establishment of Leishmania major infection transmitted by sand fly bite. PLoS Pathog. 16:e1008674. doi: 10.1371/journal.ppat.1008674, PMID: 33137149 PMC7660907

[ref126] Costa C. H. Peters N. C. Maruyama S. R. De Brito E. C. Jr. Santos I. K. (2011). Vaccines for the leishmaniases: proposals for a research agenda. PLoS Negl. Trop. Dis. 5:e943. doi: 10.1371/journal.pntd.0000943 21468307 PMC3066138

[ref13] Cranenburgh R. M. Hanak J. A. Williams S. G. Sherratt D. J. (2001). *Escherichia coli* strains that allow antibiotic-free plasmid selection and maintenance by repressor titration. Nucleic Acids Res. 29, 26e–226e. doi: 10.1093/nar/29.5.e26 PMC29739 11222777

[ref14] Damerow S. Hoppe C. Bandini G. Zarnovican P. Buettner F. F. Luder C. G.. (2015). Depletion of UDP-glucose and UDP-galactose using a Degron system leads to growth cessation of *Leishmania major*. PLoS Negl. Trop. Dis. 9:e0004205. doi: 10.1371/journal.pntd.0004205, PMID: 26529232 PMC4631452

[ref15] Darquet A. M. Cameron B. Wils P. Scherman D. Crouzet J. (1997). A new DNA vehicle for nonviral gene delivery: supercoiled minicircle. Gene Ther. 4, 1341–1349. doi: 10.1038/sj.gt.3300540, PMID: 9472558

[ref16] Davarpanah E. Seyed N. Bahrami F. Rafati S. Safaralizadeh R. Taheri T. (2020). *Lactococcus lactis* expressing sand fly PpSP15 salivary protein confers long-term protection against *Leishmania major* in BALB/c mice. PLoS Negl. Trop. Dis. 14:e0007939. doi: 10.1371/journal.pntd.0007939, PMID: 31899767 PMC6941807

[ref17] de Paiva R. M. Grazielle-Silva V. Cardoso M. S. Nakagaki B. N. Mendonca-Neto R. P. Canavaci A. M.. (2015). Amastin knockdown in *Leishmania braziliensis* affects parasite-macrophage interaction and results in impaired viability of intracellular amastigotes. PLoS Pathog. 11:e1005296. doi: 10.1371/journal.ppat.1005296, PMID: 26641088 PMC4671664

[ref18] de Souza Carmo E. V. Katz S. Barbieri C. L. (2010). Neutrophils reduce the parasite burden in *Leishmania (Leishmania)* amazonensis-infected macrophages. PLoS One 5:e13815. doi: 10.1371/journal.pone.0013815, PMID: 21082032 PMC2972777

[ref19] Dean S. Sunter J. Wheeler R. J. Hodkinson I. Gluenz E. Gull K. (2015). A toolkit enabling efficient, scalable and reproducible gene tagging in trypanosomatids. Open Biol. 5:140197. doi: 10.1098/rsob.140197, PMID: 25567099 PMC4313374

[ref20] Dey R. Joshi A. B. Oliveira F. Pereira L. Guimaraes-Costa A. B. Serafim T. D.. (2018). Gut microbes egested during bites of infected sand flies augment severity of Leishmaniasis via Inflammasome-derived IL-1beta. Cell Host Microbe 23, 134–143.e6. doi: 10.1016/j.chom.2017.12.002, PMID: 29290574 PMC5832060

[ref21] Dietz W. M. Skinner N. E. Hamilton S. E. Jund M. D. Heitfeld S. M. Litterman A. J.. (2013). Minicircle DNA is superior to plasmid DNA in eliciting antigen-specific CD8+ T-cell responses. Mol. Ther. 21, 1526–1535. doi: 10.1038/mt.2013.85, PMID: 23689601 PMC3734653

[ref22] Dinc R. (2022). Leishmania vaccines: the current situation with its promising aspect for the future. Korean J. Parasitol. 60, 379–391. doi: 10.3347/kjp.2022.60.6.379, PMID: 36588414 PMC9806502

[ref23] Dong G. Filho A. L. Olivier M. (2019). Modulation of host-pathogen communication by extracellular vesicles (EVs) of the protozoan parasite *Leishmania*. Front. Cell. Infect. Microbiol. 9:100. doi: 10.3389/fcimb.2019.00100, PMID: 31032233 PMC6470181

[ref24] Dong G. Wagner V. Minguez-Menendez A. Fernandez-Prada C. Olivier M. (2021). Extracellular vesicles and leishmaniasis: current knowledge and promising avenues for future development. Mol. Immunol. 135, 73–83. doi: 10.1016/j.molimm.2021.04.003, PMID: 33873096

[ref25] Duncan S. M. Jones N. G. Mottram J. C. (2017). Recent advances in Leishmania reverse genetics: manipulating a manipulative parasite. Mol. Biochem. Parasitol. 216, 30–38. doi: 10.1016/j.molbiopara.2017.06.005, PMID: 28629934

[ref26] Eusebio D. Neves A. R. Costa D. Biswas S. Alves G. Cui Z.. (2021). Methods to improve the immunogenicity of plasmid DNA vaccines. Drug Discov. Today 26, 2575–2592. doi: 10.1016/j.drudis.2021.06.008, PMID: 34214667

[ref27] Gary E. N. Weiner D. B. (2020). DNA vaccines: prime time is now. Curr. Opin. Immunol. 65, 21–27. doi: 10.1016/j.coi.2020.01.006, PMID: 32259744 PMC7195337

[ref28] Glennie N. D. Scott P. (2016). Memory T cells in cutaneous leishmaniasis. Cell. Immunol. 309, 50–54. doi: 10.1016/j.cellimm.2016.07.010, PMID: 27493096 PMC5127769

[ref29] Glennie N. D. Yeramilli V. A. Beiting D. P. Volk S. W. Weaver C. T. Scott P. (2015). Skin-resident memory CD4+ T cells enhance protection against Leishmania major infection. J. Exp. Med. 212, 1405–1414. doi: 10.1084/jem.20142101, PMID: 26216123 PMC4548053

[ref30] Goh S. Good L. (2008). Plasmid selection in *Escherichia coli* using an endogenous essential gene marker. BMC Biotechnol. 8:61. doi: 10.1186/1472-6750-8-61, PMID: 18694482 PMC2527308

[ref31] Gomes R. Teixeira C. Oliveira F. Lawyer P. G. Elnaiem D. E. Meneses C.. (2012). KSAC, a defined Leishmania antigen, plus adjuvant protects against the virulence of *L. major* transmitted by its natural vector *Phlebotomus duboscqi*. PLoS Negl. Trop. Dis. 6:e1610. doi: 10.1371/journal.pntd.0001610, PMID: 22509423 PMC3317914

[ref32] Gracey Maniar L. E. Maniar J. M. Chen Z. Y. Lu J. Fire A. Z. Kay M. A. (2013). Minicircle DNA vectors achieve sustained expression reflected by active chromatin and transcriptional level. Mol. Ther. 21, 131–138. doi: 10.1038/mt.2012.244, PMID: 23183534 PMC3538319

[ref33] Grybchuk D. Macedo D. H. Kleschenko Y. Kraeva N. Lukashev A. N. Bates P. A.. (2020). The first non-LRV RNA virus in *Leishmania*. Viruses 12:168. doi: 10.3390/v12020168, PMID: 32024293 PMC7077295

[ref34] Guimaraes-Costa A. B. Shannon J. P. Waclawiak I. Oliveira J. Meneses C. de Castro W.. (2021). A sand fly salivary protein acts as a neutrophil chemoattractant. Nat. Commun. 12:3213. doi: 10.1038/s41467-021-23002-5, PMID: 34050141 PMC8163758

[ref35] Gurunathan S. Sacks D. L. Brown D. R. Reiner S. L. Charest H. Glaichenhaus N.. (1997). Vaccination with DNA encoding the immunodominant LACK parasite antigen confers protective immunity to mice infected with *Leishmania major*. J. Exp. Med. 186, 1137–1147. doi: 10.1084/jem.186.7.1137, PMID: 9314562 PMC2199076

[ref36] Gurunathan S. Wu C. Y. Freidag B. L. Seder R. A. (2000). DNA vaccines: a key for inducing long-term cellular immunity. Curr. Opin. Immunol. 12, 442–447. doi: 10.1016/S0952-7915(00)00118-7, PMID: 10899026

[ref37] Haghdoust S. Noroozbeygi M. Hajimollahoseini M. Masooleh M. M. Yeganeh F. (2022). A candidate vaccine composed of live nonpathogenic Iranian lizard Leishmania mixed with chitin microparticles protects mice against *Leishmania major* infection. Acta Trop. 227:106298. doi: 10.1016/j.actatropica.2021.106298, PMID: 34971566

[ref38] Hartley M.-A. Ronet C. Zangger H. Beverley S. M. Fasel N. (2012). Leishmania RNA virus: when the host pays the toll. Front. Cell. Infect. Microbiol. 2:99. doi: 10.3389/fcimb.2012.00099 22919688 PMC3417650

[ref39] Ho W. Gao M. Li F. Li Z. Zhang X. Q. Xu X. (2021). Next-generation vaccines: nanoparticle-mediated DNA and mRNA delivery. Adv. Healthc. Mater. 10:e2001812. doi: 10.1002/adhm.202001812, PMID: 33458958 PMC7995055

[ref40] Hohman L. S. Carneiro M. B. Kratofil R. M. Peters N. C. (2021). Effector function prior to establishment of the phagosomal pathogen niche is required for protective CD4+ T cell-mediated immunity against *Leishmania*. J. Immunol. 206:16.29. doi: 10.4049/jimmunol.206.Supp.16.29

[ref41] Hohman L. S. Mou Z. Carneiro M. B. Ferland G. Kratofil R. M. Kubes P.. (2021). Protective CD4+ Th1 cell-mediated immunity is reliant upon execution of effector function prior to the establishment of the pathogen niche. PLoS Pathog. 17:e1009944. doi: 10.1371/journal.ppat.1009944, PMID: 34543348 PMC8483310

[ref42] Hohman L. S. Peters N. C. (2019). CD4(+) T cell-mediated immunity against the Phagosomal pathogen Leishmania: implications for vaccination. Trends Parasitol. 35, 423–435. doi: 10.1016/j.pt.2019.04.002, PMID: 31080088

[ref43] Ishemgulova A. Kraeva N. Faktorová D. Podesvova L. Lukes J. Yurchenko V. (2016). T7 polymerase-driven transcription is downregulated in metacyclic promastigotes and amastigotes of *Leishmania mexicana*. Folia Parasitol. 63:1. doi: 10.14411/fp.2016.016 27311571

[ref44] Ismail N. Karmakar S. Bhattacharya P. Sepahpour T. Takeda K. Hamano S.. (2022). Leishmania major Centrin gene-deleted parasites generate skin resident memory T-cell immune response analogous to Leishmanization. Front. Immunol. 13:864031. doi: 10.3389/fimmu.2022.864031, PMID: 35419001 PMC8996177

[ref45] Jiang Y. Gao X. Xu K. Wang J. Huang H. Shi C.. (2019). A novel Cre recombinase-mediated *in vivo* Minicircle DNA (CRIM) vaccine provides partial protection against Newcastle disease virus. Appl. Environ. Microbiol. 85:e00407-19. doi: 10.1128/AEM.00407-19, PMID: 31053588 PMC6606863

[ref46] Kamhavi S. Valenzuela J. G. Coutinho-Abreu I. V. Brodskyn C. I. Role of sand fly saliva on Leishmania-infection and the potential of vector salivary proteins as vaccines. Skin and arthropod vectors. Academic Press, London. (2018), 96–119.

[ref47] Kamhawi S. (2000). The biological and immunomodulatory properties of sand fly saliva and its role in the establishment of *Leishmania infections*. Microbes Infect. 2, 1765–1773. doi: 10.1016/S1286-4579(00)01331-9, PMID: 11137049

[ref48] Karmakar S. Volpedo G. Zhang W. W. Lypaczewski P. Ismail N. Oliveira F.. (2022). Centrin-deficient Leishmania mexicana confers protection against Old World visceral leishmaniasis. NPJ Vaccines. 7:157. doi: 10.1038/s41541-022-00574-x, PMID: 36463228 PMC9719514

[ref49] Katebi A. Gholami E. Taheri T. Zahedifard F. Habibzadeh S. Taslimi Y.. (2015). *Leishmania tarentolae* secreting the sand fly salivary antigen PpSP15 confers protection against *Leishmania* major infection in a susceptible BALB/c mice model. Mol. Immunol. 67, 501–511. doi: 10.1016/j.molimm.2015.08.001 26298575

[ref50] Kaye P. M. Mohan S. Mantel C. Malhame M. Revill P. Le Rutte E.. (2021). Overcoming roadblocks in the development of vaccines for leishmaniasis. Expert Rev. Vaccines 20, 1419–1430. doi: 10.1080/14760584.2021.1990043, PMID: 34727814 PMC9844205

[ref51] Keshavarzian N. Noroozbeygi M. Haji Molla Hoseini M. Yeganeh F. (2020). Evaluation of Leishmanization using Iranian lizard Leishmania mixed with CpG-ODN as a candidate vaccine against experimental murine Leishmaniasis. Front. Immunol. 11:1725. doi: 10.3389/fimmu.2020.01725, PMID: 33193290 PMC7645074

[ref52] Kutzler M. A. Weiner D. B. (2008). DNA vaccines: ready for prime time? Nat. Rev. Genet. 9, 776–788. doi: 10.1038/nrg2432, PMID: 18781156 PMC4317294

[ref53] Lajevardi M. S. Gholami E. Taheri T. Sarvnaz H. Habibzadeh S. Seyed N.. (2022). *Leishmania tarentolae* as potential live vaccine co-expressing distinct salivary gland proteins against experimental cutaneous leishmaniasis in BALB/c mice model. Front. Immunol. 13:895234. doi: 10.3389/fimmu.2022.895234, PMID: 35757692 PMC9226313

[ref54] Lee J. Kumar S. A. Jhan Y. Y. Bishop C. J. (2018). Engineering DNA vaccines against infectious diseases. Acta Biomater. 80, 31–47. doi: 10.1016/j.actbio.2018.08.033, PMID: 30172933 PMC7105045

[ref55] Lestinova T. Rohousova I. Sima M. de Oliveira C. I. Volf P. (2017). Insights into the sand fly saliva: blood-feeding and immune interactions between sand flies, hosts, and *Leishmania*. PLoS Negl. Trop. Dis. 11:e0005600. doi: 10.1371/journal.pntd.0005600, PMID: 28704370 PMC5509103

[ref56] Li F. Cheng L. Murphy C. M. Reszka-Blanco N. J. Wu Y. Chi L.. (2016). Minicircle HBV cccDNA with a Gaussia luciferase reporter for investigating HBV cccDNA biology and developing cccDNA-targeting drugs. Sci. Rep. 6:36483. doi: 10.1038/srep36483 27819342 PMC5098228

[ref57] Li L. Petrovsky N. (2016). Molecular mechanisms for enhanced DNA vaccine immunogenicity. Expert Rev. Vaccines 15, 313–329. doi: 10.1586/14760584.2016.1124762, PMID: 26707950 PMC4955855

[ref58] Lim M. Badruddoza A. Z. M. Firdous J. Azad M. Mannan A. Al-Hilal T. A.. (2020). Engineered Nanodelivery systems to improve DNA vaccine technologies. Pharmaceutics 12:30. doi: 10.3390/pharmaceutics12010030, PMID: 31906277 PMC7022884

[ref59] Liu M. A. (2011). DNA vaccines: an historical perspective and view to the future. Immunol. Rev. 239, 62–84. doi: 10.1111/j.1600-065X.2010.00980.x, PMID: 21198665

[ref60] Lopes A. Vandermeulen G. Preat V. (2019). Cancer DNA vaccines: current preclinical and clinical developments and future perspectives. J. Exp. Clin. Cancer Res. 38:146. doi: 10.1186/s13046-019-1154-7, PMID: 30953535 PMC6449928

[ref61] Louis L. Clark M. Wise M. C. Glennie N. Wong A. Broderick K.. (2019). Intradermal synthetic DNA vaccination generates *Leishmania*-specific T cells in the skin and protection against *Leishmania major*. Infect. Immun. 87:e00227-19. doi: 10.1128/IAI.00227-19, PMID: 31182618 PMC6652766

[ref62] Lu J. Zhang F. Xu S. Fire A. Z. Kay M. A. (2012). The extragenic spacer length between the 5′ and 3′ ends of the transgene expression cassette affects transgene silencing from plasmid-based vectors. Mol. Ther. 20, 2111–2119. doi: 10.1038/mt.2012.65, PMID: 22565847 PMC3498813

[ref63] Lukacs G. L. Haggie P. Seksek O. Lechardeur D. Freedman N. Verkman A. S. (2000). Size-dependent DNA mobility in cytoplasm and nucleus. J. Biol. Chem. 275, 1625–1629. doi: 10.1074/jbc.275.3.1625, PMID: 10636854

[ref64] Luke J. Carnes A. E. Hodgson C. P. Williams J. A. (2009). Improved antibiotic-free DNA vaccine vectors utilizing a novel RNA based plasmid selection system. Vaccine 27, 6454–6459. doi: 10.1016/j.vaccine.2009.06.017, PMID: 19559109 PMC2767433

[ref65] Madeira da Silva L. Beverley S. M. (2010). Expansion of the target of rapamycin (TOR) kinase family and function in Leishmania shows that TOR3 is required for acidocalcisome biogenesis and animal infectivity. Proc. Natl. Acad. Sci. USA 107, 11965–11970. doi: 10.1073/pnas.1004599107, PMID: 20551225 PMC2900677

[ref66] Mairhofer J. Cserjan-Puschmann M. Striedner G. Nobauer K. Razzazi-Fazeli E. Grabherr R. (2010). Marker-free plasmids for gene therapeutic applications--lack of antibiotic resistance gene substantially improves the manufacturing process. J. Biotechnol. 146, 130–137. doi: 10.1016/j.jbiotec.2010.01.025, PMID: 20138928

[ref67] Marie C. Richard M. Vandermeulen G. Quiviger M. Préat V. Scherman D. (2008). pFAR plasmids: new eukaryotic expression vectors for gene therapy, devoid of antibiotic resistance markers. Nat. Preced.:1. doi: 10.1038/npre.2008.2395.1

[ref68] Marie C. Vandermeulen G. Quiviger M. Richard M. Preat V. Scherman D. (2010). pFARs, plasmids free of antibiotic resistance markers, display high-level transgene expression in muscle, skin and tumour cells. J. Gene Med. 12, 323–332. doi: 10.1002/jgm.1441, PMID: 20209487

[ref69] Masoudzadeh N. Mizbani A. Rafati S. (2020a). Transcriptomic profiling in cutaneous Leishmaniasis patients. Expert Rev. Proteomics 17, 533–541. doi: 10.1080/14789450.2020.1812390, PMID: 32886890

[ref70] Masoudzadeh N. Ostensson M. Persson J. Mashayekhi Goyonlo V. Agbajogu C. Taslimi Y.. (2020b). Molecular signatures of anthroponotic cutaneous leishmaniasis in the lesions of patients infected with *Leishmania tropica*. Sci. Rep. 10:16198. doi: 10.1038/s41598-020-72671-7, PMID: 33004861 PMC7529897

[ref71] McCall L. I. El Aroussi A. Choi J. Y. Vieira D. F. De Muylder G. Johnston J. B.. (2015). Targeting Ergosterol biosynthesis in *Leishmania donovani*: essentiality of sterol 14 alpha-demethylase. PLoS Negl. Trop. Dis. 9:e0003588. doi: 10.1371/journal.pntd.0003588, PMID: 25768284 PMC4359151

[ref72] McCluskie M. J. Weeratna R. D. Davis H. L. (2000). The role of CpG in DNA vaccines. Springer Semin. Immunopathol. 22, 125–132. doi: 10.1007/s002810000014 10944807

[ref73] Mendez S. Gurunathan S. Kamhawi S. Belkaid Y. Moga M. A. Skeiky Y. A.. (2001). The potency and durability of DNA-and protein-based vaccines against *Leishmania major* evaluated using low-dose, intradermal challenge. J. Immunol. 166, 5122–5128. doi: 10.4049/jimmunol.166.8.5122 11290794

[ref74] Mendoza-Roldan J. A. Latrofa M. S. Iatta R. Manoj R R. S. Panarese R. Annoscia G.. (2021). Detection of *Leishmania tarentolae* in lizards, sand flies and dogs in southern Italy, where *Leishmania* infantum is endemic: hindrances and opportunities. Parasit. Vectors 14, 1–12. doi: 10.1186/s13071-021-04973-2 34493323 PMC8423600

[ref75] Mendoza-Roldan J. A. Votypka J. Bandi C. Epis S. Modry D. Ticha L.. (2022). *Leishmania tarentolae*: a new frontier in the epidemiology and control of the leishmaniases. Transbound. Emerg. Dis. 69, e1326–e1337. doi: 10.1111/tbed.14660, PMID: 35839512 PMC9804434

[ref77] Moreira P. O. L. Nogueira P. M. Monte-Neto R. L. (2023). Next-generation Leishmanization: revisiting molecular targets for selecting genetically engineered live-attenuated Leishmania. Microorganisms. 11:1043. doi: 10.3390/microorganisms11041043 37110466 PMC10145799

[ref78] Munye M. M. Tagalakis A. D. Barnes J. L. Brown R. E. McAnulty R. J. Howe S. J.. (2016). Minicircle DNA provides enhanced and prolonged transgene expression following airway gene transfer. Sci. Rep. 6:23125. doi: 10.1038/srep23125, PMID: 26975732 PMC4792149

[ref79] Mwau M. Cebere I. Sutton J. Chikoti P. Winstone N. Wee E. G.. (2004). A human immunodeficiency virus 1 (HIV-1) clade a vaccine in clinical trials: stimulation of HIV-specific T-cell responses by DNA and recombinant modified vaccinia virus Ankara (MVA) vaccines in humans. J. Gen. Virol. 85, 911–919. doi: 10.1099/vir.0.19701-0, PMID: 15039533

[ref80] Noroozbeygi M. Keshavarzian N. Haji Molla Hoseini M. Haghdoust S. Yeganeh F. (2023). Comparison of the long-term and short-term protection in mouse model of *Leishmania major* infection following vaccination with live Iranian lizard Leishmania mixed with chitin microparticles. Parasite Immunol. 46:e13018. doi: 10.1111/pim.13018 37987175

[ref81] Omondi Z. N. Demir S. (2021). Bacteria composition and diversity in the gut of sand fly: impact on Leishmania and sand fly development. Int. J. Trop. Insect. Sci. 41, 25–32. doi: 10.1007/s42690-020-00184-x

[ref82] Pacheco-Fernandez T. Volpedo G. Gannavaram S. Bhattacharya P. Dey R. Satoskar A.. (2021). Revival of Leishmanization and Leishmanin. Front. Cell. Infect. Microbiol. 11:639801. doi: 10.3389/fcimb.2021.639801, PMID: 33816344 PMC8010169

[ref83] Pandey S. C. Kumar A. Samant M. (2020). Genetically modified live attenuated vaccine: a potential strategy to combat visceral leishmaniasis. Parasite Immunol. 42:e12732. doi: 10.1111/pim.12732 32418227

[ref84] Pang X. Ma F. Zhang P. Zhong Y. Zhang J. Wang T.. (2017). Treatment of human B-cell lymphomas using Minicircle DNA vector expressing anti-CD3/CD20 in a mouse model. Hum. Gene Ther. 28, 216–225. doi: 10.1089/hum.2016.122 27802782

[ref85] Passelli K. Billion O. Tacchini-Cottier F. (2021). The impact of neutrophil recruitment to the skin on the pathology induced by *Leishmania* infection. Front. Immunol. 12:649348. doi: 10.3389/fimmu.2021.649348, PMID: 33732265 PMC7957080

[ref86] Peters N. C. Bertholet S. Lawyer P. G. Charmoy M. Romano A. Ribeiro-Gomes F. L.. (2012). Evaluation of recombinant Leishmania polyprotein plus glucopyranosyl lipid a stable emulsion vaccines against sand fly-transmitted *Leishmania* major in C57BL/6 mice. J. Immunol. 189, 4832–4841. doi: 10.4049/jimmunol.1201676, PMID: 23045616 PMC3596879

[ref87] Peters N. C. Egen J. G. Secundino N. Debrabant A. Kimblin N. Kamhawi S.. (2008). *In vivo* imaging reveals an essential role for neutrophils in leishmaniasis transmitted by sand flies. Science 321, 970–974. doi: 10.1126/science.1159194, PMID: 18703742 PMC2606057

[ref88] Peters N. C. Kimblin N. Secundino N. Kamhawi S. Lawyer P. Sacks D. L. (2009). Vector transmission of leishmania abrogates vaccine-induced protective immunity. PLoS Pathog. 5:e1000484. doi: 10.1371/journal.ppat.1000484, PMID: 19543375 PMC2691580

[ref89] Peters N. C. Pagan A. J. Lawyer P. G. Hand T. W. Henrique Roma E. Stamper L. W.. (2014). Chronic parasitic infection maintains high frequencies of short-lived Ly6C+CD4+ effector T cells that are required for protection against re-infection. PLoS Pathog. 10:e1004538. doi: 10.1371/journal.ppat.1004538, PMID: 25473946 PMC4256462

[ref90] Peters N. C. Sacks D. L. (2009). The impact of vector-mediated neutrophil recruitment on cutaneous leishmaniasis. Cell. Microbiol. 11, 1290–1296. doi: 10.1111/j.1462-5822.2009.01348.x, PMID: 19545276 PMC3431610

[ref91] Pfaffenzeller I. Mairhofer J. Striedner G. Bayer K. Grabherr R. (2006). Using ColE1-derived RNA I for suppression of a bacterially encoded gene: implication for a novel plasmid addiction system. Biotechnol. J. 1, 675–681. doi: 10.1002/biot.200600017, PMID: 16892316

[ref92] Plotkin S. (2014). History of vaccination. Proc. Natl. Acad. Sci. USA 111, 12283–12287. doi: 10.1073/pnas.1400472111, PMID: 25136134 PMC4151719

[ref93] Ramos I. Alonso A. Peris A. Marcen J. M. Abengozar M. A. Alcolea P. J.. (2009). Antibiotic resistance free plasmid DNA expressing LACK protein leads towards a protective Th1 response against *Leishmania* infantum infection. Vaccine 27, 6695–6703. doi: 10.1016/j.vaccine.2009.08.091, PMID: 19747996

[ref94] Raymond F. Boisvert S. Roy G. Ritt J. F. Legare D. Isnard A.. (2012). Genome sequencing of the lizard parasite *Leishmania tarentolae* reveals loss of genes associated to the intracellular stage of human pathogenic species. Nucleic Acids Res. 40, 1131–1147. doi: 10.1093/nar/gkr834, PMID: 21998295 PMC3273817

[ref95] Ronet C. Passelli K. Charmoy M. Scarpellino L. Myburgh E. Hauyon La Torre Y.. (2019). TLR2 signaling in skin nonhematopoietic cells induces early neutrophil recruitment in response to Leishmania major infection. J. Invest. Dermatol. 139, 1318–1328. doi: 10.1016/j.jid.2018.12.012, PMID: 30594488 PMC8024985

[ref96] Rosazza C. Escoffre J.-M. Zumbusch A. Rols M.-P. (2011). The actin cytoskeleton has an active role in the electrotransfer of plasmid DNA in mammalian cells. Mol. Ther. 19, 913–921. doi: 10.1038/mt.2010.303, PMID: 21343915 PMC3098633

[ref97] Rossi M. Fasel N. (2018). The criminal association of Leishmania parasites and viruses. Curr. Opin. Microbiol. 46, 65–72. doi: 10.1016/j.mib.2018.07.005, PMID: 30096485

[ref98] Rozkov A. Avignone-Rossa C. A. Ertl P. F. Jones P. O'Kennedy R. D. Smith J. J.. (2004). Characterization of the metabolic burden on *Escherichia coli* DH1 cells imposed by the presence of a plasmid containing a gene therapy sequence. Biotechnol. Bioeng. 88, 909–915. doi: 10.1002/bit.20327, PMID: 15532038

[ref99] Sacks D. L. (2014). Vaccines against tropical parasitic diseases: a persisting answer to a persisting problem. Nat. Immunol. 15, 403–405. doi: 10.1038/ni.2853 24747701 PMC4814932

[ref100] Sainz M. de la Maza O. (2014). Leishmaniasis transmission biology: Role of promastigote secretory gel as a transmission determinant London School of Hygiene & Tropical Medicine.

[ref101] Salisbury J. L. (2004). Centrosomes: Sfi1p and centrin unravel a structural riddle. Curr. Biol. 14, R27–R29. doi: 10.1016/j.cub.2003.12.019, PMID: 14711432

[ref102] Saljoughian N. Taheri T. Zahedifard F. Taslimi Y. Doustdari F. Bolhassani A.. (2013). Development of novel prime-boost strategies based on a tri-gene fusion recombinant *L. tarentolae* vaccine against experimental murine visceral leishmaniasis. PLoS Negl. Trop. Dis. 7:e2174. doi: 10.1371/journal.pntd.0002174, PMID: 23638195 PMC3630202

[ref103] Sangare L. Manhart L. Zehrung D. Wang C. C. (2009). Intradermal hepatitis B vaccination: a systematic review and meta-analysis. Vaccine 27, 1777–1786. doi: 10.1016/j.vaccine.2009.01.043, PMID: 19200451

[ref104] Santos R. Silva G. L. A. Santos E. V. Duncan S. M. Mottram J. C. Damasceno J. D.. (2017). A DiCre recombinase-based system for inducible expression in *Leishmania major*. Mol. Biochem. Parasitol. 216, 45–48. doi: 10.1016/j.molbiopara.2017.06.006, PMID: 28629935

[ref105] Schindler S. E. McCall J. G. Yan P. Hyrc K. L. Li M. Tucker C. L.. (2015). Photo-activatable Cre recombinase regulates gene expression *in vivo*. Sci. Rep. 5:13627. doi: 10.1038/srep13627, PMID: 26350769 PMC4563371

[ref106] Scott P. (2020). Long-lived skin-resident memory T cells contribute to concomitant immunity in cutaneous Leishmaniasis. Cold Spring Harb. Perspect. Biol. 12:a038059. doi: 10.1101/cshperspect.a038059 32839202 PMC7528853

[ref107] Selvapandiyan A. Debrabant A. Duncan R. Muller J. Salotra P. Sreenivas G.. (2004). Centrin gene disruption impairs stage-specific basal body duplication and cell cycle progression in Leishmania. J. Biol. Chem. 279, 25703–25710. doi: 10.1074/jbc.M402794200, PMID: 15084606

[ref108] Selvapandiyan A. Duncan R. Debrabant A. Bertholet S. Sreenivas G. Negi N. S.. (2001). Expression of a mutant form of *Leishmania donovani* centrin reduces the growth of the parasite. J. Biol. Chem. 276, 43253–43261. doi: 10.1074/jbc.M106806200, PMID: 11544261

[ref109] Serafim T. D. Coutinho-Abreu I. V. Dey R. Kissinger R. Valenzuela J. G. Oliveira F.. (2021). Leishmaniasis: the act of transmission. Trends Parasitol. 37, 976–987. doi: 10.1016/j.pt.2021.07.003, PMID: 34389215

[ref110] Seyed N. Peters N. C. Rafati S. (2018). Translating observations from *Leishmanization* into non-living vaccines: the potential of dendritic cell-based vaccination strategies against Leishmania. Front. Immunol. 9:1227. doi: 10.3389/fimmu.2018.01227, PMID: 29922288 PMC5996938

[ref111] Seyed N. Rafati S. (2021). Th1 concomitant immune response mediated by IFN-gamma protects against sand fly delivered *Leishmania* infection: implications for vaccine design. Cytokine 147:155247. doi: 10.1016/j.cyto.2020.155247, PMID: 32873468

[ref112] Silvestre R. Cordeiro-da-Silva A. Ouaissi A. (2008). Live attenuated Leishmania vaccines: a potential strategic alternative. Arch. Immunol. Ther. Exp. 56, 123–126. doi: 10.1007/s00005-008-0010-9, PMID: 18373245

[ref113] Singh K. Kaviraj S. Karmakar S. Singh S. (2022). Vaccine for leishmaniasis: new era of CRISPR generated live attenuated dermotropic Leishmania. J Cell Mol Immunol. 1, 12–16. doi: 10.46439/immunol.1.004

[ref114] Spath G. F. Lye L.-F. Segawa H. Sacks D. L. Turco S. J. Beverley S. M. (2003). Persistence without pathology in phosphoglycan-deficient Leishmania major. Science 301, 1241–1243. doi: 10.1126/science.1087499 12947201

[ref115] Spath G. F. Lye L. F. Segawa H. Turco S. J. Beverley S. M. (2004). Identification of a compensatory mutant (lpg2-REV) of Leishmania major able to survive as amastigotes within macrophages without LPG2-dependent glycoconjugates and its significance to virulence and immunization strategies. Infect. Immun. 72, 3622–3627. doi: 10.1128/IAI.72.6.3622-3627.2004, PMID: 15155672 PMC415719

[ref116] Suschak J. J. Dupuy L. C. Shoemaker C. J. Six C. Kwilas S. A. Spik K. W.. (2020). Nanoplasmid vectors co-expressing innate immune agonists enhance DNA vaccines for Venezuelan equine encephalitis virus and Ebola virus. Mol. Ther. Methods Clin. Dev. 17, 810–821. doi: 10.1016/j.omtm.2020.04.009, PMID: 32296729 PMC7158766

[ref117] Suschak J. J. Williams J. A. Schmaljohn C. S. (2017). Advancements in DNA vaccine vectors, non-mechanical delivery methods, and molecular adjuvants to increase immunogenicity. Hum. Vaccin. Immunother. 13, 2837–2848. doi: 10.1080/21645515.2017.1330236, PMID: 28604157 PMC5718814

[ref118] Topuz Ata D. Hussain M. Jones M. Best J. Wiese M. Carter K. C. (2023). Immunisation with transgenic *L. tarentolae* expressing gamma glutamyl cysteine synthetase from pathogenic leishmania species protected against *L. major* and *L. donovani* infection in a murine model. Microorganisms. 11:1322. doi: 10.3390/microorganisms11051322 37317296 PMC10223578

[ref119] Uzonna J. E. Spath G. F. Beverley S. M. Scott P. (2004). Vaccination with phosphoglycan-deficient Leishmania major protects highly susceptible mice from virulent challenge without inducing a strong Th1 response. J. Immunol. 172, 3793–3797. doi: 10.4049/jimmunol.172.6.3793, PMID: 15004184

[ref120] Valenzuela J. G. Belkaid Y. Garfield M. K. Mendez S. Kamhawi S. Rowton E. D.. (2001). Toward a defined anti-Leishmania vaccine targeting vector antigens: characterization of a protective salivary protein. J. Exp. Med. 194, 331–342. doi: 10.1084/jem.194.3.331, PMID: 11489952 PMC2193460

[ref121] Vandermeulen G. Marie C. Scherman D. Preat V. (2011). New generation of plasmid backbones devoid of antibiotic resistance marker for gene therapy trials. Mol. Ther. 19, 1942–1949. doi: 10.1038/mt.2011.182, PMID: 21878901 PMC3222533

[ref122] Volpedo G. Bhattacharya P. Gannavaram S. Pacheco-Fernandez T. Oljuskin T. Dey R.. (2022). The history of live attenuated Centrin gene-deleted Leishmania vaccine candidates. Pathogens 11:431. doi: 10.3390/pathogens11040431, PMID: 35456106 PMC9025045

[ref123] Volpedo G. Huston R. H. Holcomb E. A. Pacheco-Fernandez T. Gannavaram S. Bhattacharya P.. (2021). From infection to vaccination: reviewing the global burden, history of vaccine development, and recurring challenges in global leishmaniasis protection. Expert Rev. Vaccines 20, 1431–1446. doi: 10.1080/14760584.2021.1969231, PMID: 34511000

[ref124] Wang Q. Jiang W. Chen Y. Liu P. Sheng C. Chen S.. (2014). *In vivo* electroporation of minicircle DNA as a novel method of vaccine delivery to enhance HIV-1-specific immune responses. J. Virol. 88, 1924–1934. doi: 10.1128/JVI.02757-13, PMID: 24284319 PMC3911567

[ref125] Williams J. A. (2013). Vector design for improved DNA vaccine efficacy. Safety Product. Vaccines (Basel) 1, 225–249. doi: 10.3390/vaccines1030225, PMID: 26344110 PMC4494225

[ref127] Yin W. Xiang P. Li Q. (2005). Investigations of the effect of DNA size in transient transfection assay using dual luciferase system. Anal. Biochem. 346, 289–294. doi: 10.1016/j.ab.2005.08.029, PMID: 16213455

[ref128] Zabala-Penafiel A. Todd D. Daneshvar H. Burchmore R. (2020). The potential of live attenuated vaccines against cutaneous Leishmaniasis. Exp. Parasitol. 210:107849. doi: 10.1016/j.exppara.2020.107849, PMID: 32027892

[ref129] Zhang W. W. Karmakar S. Gannavaram S. Dey R. Lypaczewski P. Ismail N.. (2020). A second generation leishmanization vaccine with a markerless attenuated Leishmania major strain using CRISPR gene editing. Nat. Commun. 11:3461. doi: 10.1038/s41467-020-17154-z, PMID: 32651371 PMC7351751

[ref130] Zhang W. W. Lypaczewski P. Matlashewski G. (2017). Optimized CRISPR-Cas9 genome editing for Leishmania and its use to target a multigene family, induce chromosomal translocation, and study DNA break repair mechanisms. mSphere 2:e00340-16. doi: 10.1128/mSphere.00340-16, PMID: 28124028 PMC5244264

[ref131] Zhang W. W. Matlashewski G. (2015). CRISPR-Cas9-mediated genome editing in *Leishmania donovani*. MBio 6:e00861. doi: 10.1128/mBio.00861-15 26199327 PMC4513079

